# Immune monitoring and risk of infection in pediatric liver transplantation: a prospective study

**DOI:** 10.3389/fimmu.2025.1605716

**Published:** 2025-06-12

**Authors:** Ricardo Cuesta-Martín de la Cámara, Laura Miguel-Berenguel, Carmen Cámara, Itsaso Losantos-García, Esteban Frauca-Remacha, Loreto Hierro-Llanillo, Gema Muñoz-Bartolo, María Dolores Lledín-Barbacho, Ana Martínez-Feito, Eduardo López-Granados, Elena Sánchez-Zapardiel

**Affiliations:** ^1^ Clinical Immunology Department, University Hospital La Paz, Madrid, Spain; ^2^ Lymphocyte Pathophysiology in Immunodeficiencies Group, La Paz Institute for Health Research (IdiPAZ), Madrid, Spain; ^3^ Medicine and Surgery Department, PhD School, Autonomous University of Madrid, Madrid, Spain; ^4^ Biostatistics Platform, La Paz Institute for Health Research (IdiPAZ), Madrid, Spain; ^5^ Paediatric Hepatology Department, University Hospital La Paz, Madrid, Spain; ^6^ European Reference Network (ERN) RARE LIVER, Madrid, Spain; ^7^ European Reference Network (ERN) TransplantChild, Madrid, Spain; ^8^ Centre for Biomedical Network Research on Rare Diseases (CIBERER U767), Madrid, Spain

**Keywords:** liver transplantation, humoral immunity, cellular immunity, immune monitoring, flow cytometry

## Abstract

**Background:**

Immune monitoring has been proposed to optimize immunosuppressive therapy in liver recipients. This study aims to describe immunological changes following liver transplantation in pediatric recipients and to identify immune markers associated with post-transplant complications.

**Methods:**

The immunological status of 95 pediatric liver recipients was prospectively assessed before transplantation and at 1, 3, 6, 9 and 12 months post-transplantation. Serum immunoglobulins (Ig) were measured by nephelometry and immunophenotype was evaluated by flow cytometry. T, B and NK lymphocyte counts were adjusted for age using standard reference ranges.

**Results:**

Graft rejection, post-transplant lymphoproliferative disorder and autoimmune hepatitis was diagnosed in 6%, 2% and 0% patients, respectively. Early infections affected 43% patients, while late infections occurred in 17%, 24%, 10% and 9% recipients at each follow-up interval. Baseline immune dysregulation primarily involved the cellular compartment, with 78% recipients showing lymphopenia. Lymphocyte subpopulation scores improved following liver transplantation, with CD4^+^ score normalizing by month 1 and CD8^+^, CD19^+^ and NK scores by month 6. First-month IgG hypogammaglobulinemia, observed in 20% recipients, resolved completely at month 12. First-month T-cell lymphopenia (CD3^+^ hazard ratio [HR] 2.48, p=0.005; CD8^+^ HR 2.38, p=0.008) and hypogammaglobulinemia (IgG HR 2.18, p=0.036; IgA HR 2.40, p=0.011; IgM HR 2.61, p=0.006) were associated with higher risk of late infections. In multivariate analysis, only CD3^+^ T-cell lymphopenia remained a significant predictor (HR 2.13, p=0.030).

**Conclusions:**

Baseline immune dysregulation resolved within the first months post-transplantation. Early infections were unrelated to immune markers, while late infections were associated with CD3^+^ T-cell lymphopenia and hypogammaglobulinemia.

## 1 Introduction

Liver transplantation (LT) remains the most effective treatment for end-stage liver disease (1). Advances in immunosuppressive therapies and surgical techniques have improved survival rates, both in adults (72-73%) (2, 3) and children (73-94%) (4, 5). However, the precise tailoring of immunosuppressive treatments for each recipient remains challenging. Striking the optimal balance between minimizing the risk of rejection and avoiding complications related to immunosuppressive drugs remains crucial (6). Among these complications, infections are the leading cause of mortality in pediatric LT recipients (4.1%) (7).

Currently, clinical practice relies primarily on pharmacokinetics to estimate immunosuppression, but this approach is often insufficient in pediatric LT (8). New strategies, including pharmacogenomics, immune biomarkers, cellular therapy, tolerance induction and alternative immunosuppressants, show promise for managing narrow therapeutic range drugs (9). Hence, immune monitoring has been proposed as a valuable tool to predict immunological and infectious complications after LT (10).

In LT humoral immune responses are monitored by the presence of donor-specific antibodies, which are often a contraindication for immunosuppression weaning (11). However, there are no standardized techniques to measure cellular responses against infections and/or malignancies. Specific T-cell responses have been proposed as biomarkers for predicting post-transplant lymphoproliferative disorder (PTLD) (12). Our previous study evaluated this approach in the pediatric LT setting, to identify patients with inadequate control of Epstein-Barr virus (EBV) infection (13).

In recent years, new follow-up strategies combining both humoral and cellular immunity in LT have been explored (14–16). Fukui et al. studied 82 adult liver recipients, finding that low serum complement 3 (C3) levels before and one month after transplantation predicted 90-day mortality (14). Previously, Iovino et al. found that liver recipients who develop infections had lower immunoglobulin G (IgG) levels at day 3 post-transplantation and higher CD64 monocyte counts at day 7 (15). Similarly, Carbone et al. had observed that liver recipients at higher infection risk had baseline hypocomplementemia C3 and hipergammablobulinemia IgG, but showed reduced IgG levels by day 7 post-transplantation (16).

While those studies focused on adults, research on immune changes in pediatric LT is limited (8), as studying immunity in children is challenging due to age-related effects on T- and B-cell number and function, influencing their susceptibility to infections and other complications (17). However, epidemiological observations (18) suggest that children exhibit more favorable outcomes than adults when confronted with viruses like EBV and Severe Acute Respiratory Syndrome Coronavirus 2, likely due to their robust innate immune responses, characterized by more active natural killer (NK) and NKT cells, as well as increased regulatory T cells (Tregs).

Given these differences, it is crucial to translate this understanding into the context of immunosuppression in pediatric LT. This prospective study aims to define humoral and cellular immunity changes before LT and up to one year after the procedure in a cohort of pediatric recipients, considering age-related variations. Additionally, we seek to identify immune markers associated with the risk of clinically relevant infections, autoimmunity, PTLD and rejection events.

## 2 Methods

### 2.1 Patients and study design

**Figure 1:**
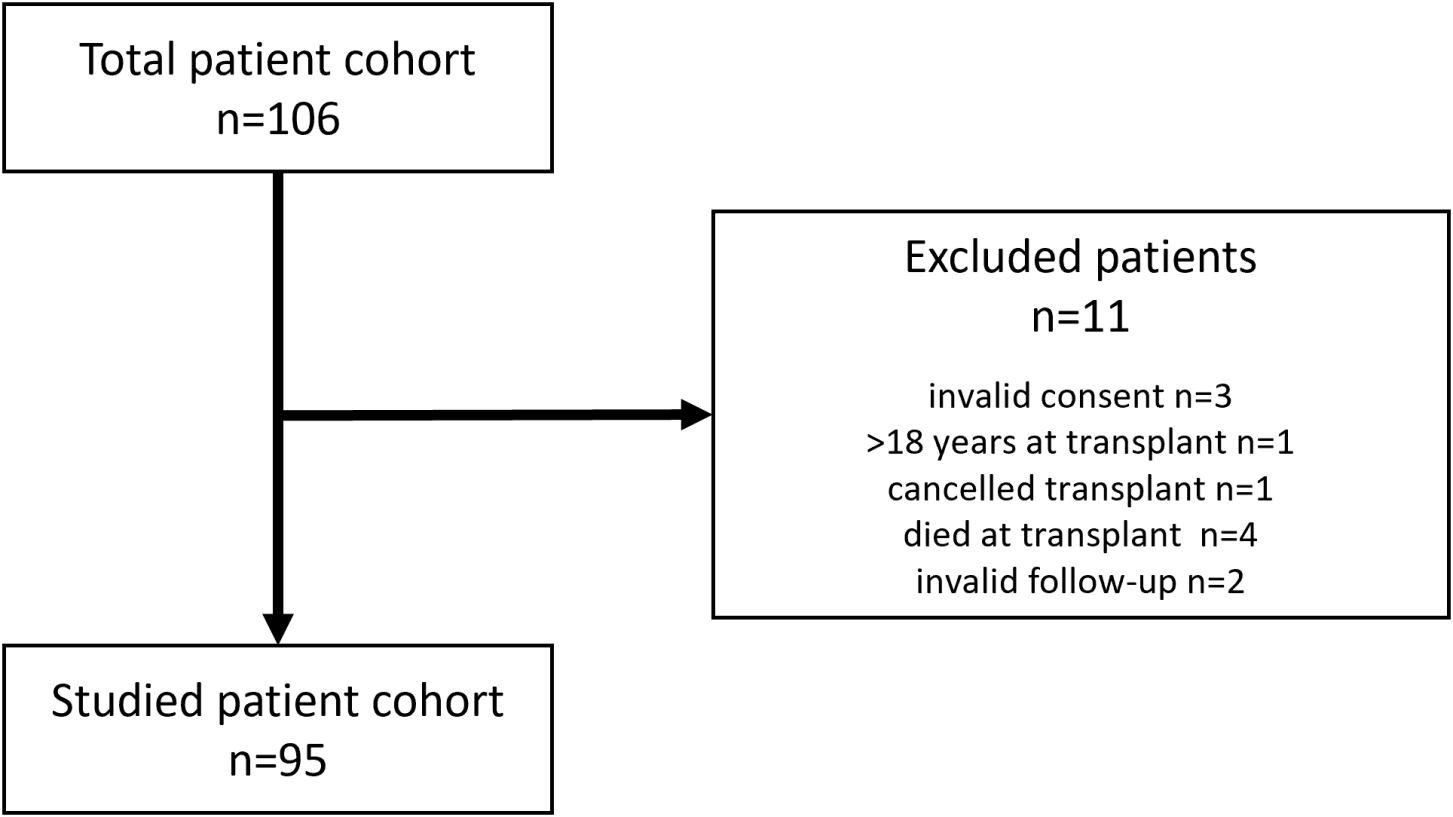
Flowchart for the inclusion of the pediatric liver transplanted patient cohort (n=95).

Our prospective study included 106 pediatric patients from University Hospital La Paz, who received a liver graft between January 2019 and December 2023. All patients gave informed consent, approved by the ethics committee of our institution (reference PI-4000). Eleven patients were withdrawn from the study (**Figure 1**), resulting in a final cohort of 95 patients. Transplant indication was categorized in five groups (**Table 1**), according to Díaz Fernandez et al. (19).

**Table 1:** Epidemiologic and clinical features in a cohort of pediatric liver recipients, further categorized as early/late infected and non-infected patients.

Characteristics	Total (n=95)	Early infection		p-value	Late infection		p-value
		No (n=54)	Yes (n=41)		No (n=56)	Yes (n=39)	
Sex, n (%)				0.540			1.000
Male	50 (53)	30 (59)	20 (49)		29 (52)	21 (54)	
Female	45 (47)	24 (41)	21 (51)		27 (48)	18 (46)	
Age at transplantation, months (IQR)	16 (7-88)	60 (13-151)	13 (8-36)	0.001	60 (17-120)	14 (10-24)	<0.001
Type of donor, n (%)				0.001			0.004
Deceased donor - split graft	39 (41)	14 (24)	25 (61)		17 (30)	22 (56)	
Deceased donor - reduced graft	23 (24)	13 (24)	10 (24)		17 (30)	6 (15)	
Deceased donor - whole graft	22 (23)	19 (17)	3 (7)		18 (32)	4 (10)	
Living donor	11 (12)	8 (15)	3 (7)		4 (7)	7 (18)	
ABO compatibility, n (%)				0.233			0.696
Compatible	88 (93)	52 (96)	36 (88)		51 (91)	37 (95)	
Incompatible	7 (7)	2 (4)	5 (12)		5 (9)	2 (5)	
Indication for transplantation, n (%)				0.489			0.179
Cholestasis/biliary atresia	60 (63)	33 (61)	27 (66)		32 (57)	28 (71)	
Metabolic diseases	12 (13)	6 (11)	6 (15)		9 (16)	3 (8)	
Liver tumours	11 (12)	9 (17)	2 (5)		9 (16)	2 (5)	
Cirrhosis (other)	8 (8)	4 (7)	4 (10)		5 (9)	3 (8)	
Severe acute liver failure	4 (4)	2 (4)	2 (5)		1 (2)	3 (8)	
Type of transplantation, n (%)				0.231			1.000
Hepatic	89 (94)	49 (81)	40 (98)		52 (93)	37 (95)	
Combined	6 (6)	5 (9)	1 (2)		4 (7)	2 (5)	
Transplant number, n (%)				0.727			0.733
First	86 (91)	48 (89)	38 (93)		50 (89)	36 (92)	
Second	9 (9)	6 (11)	3 (7)		6 (11)	3 (8)	
Induction treatment, n (%)				0.504			0.066
TAC+CE+BSX	92 (97)	51 (94)	41 (100)		56 (100)	36 (92)	
TAC+CE+BSX+MMF	2 (2)	2 (4)	0 (0)		0 (0)	2 (5)	
TAC+CE+BSX+QT	1 (1)	1 (2)	0 (0)		0 (0)	1 (3)	
Maintenance treatment at 1M, n (%)				N/A			0.938
TAC+CE	76 (80)	N/A	N/A		44 (78)	32 (82)	
TAC+CE+BSX+MMF	16 (17)				10 (18)	6 (15)	
TAC+CE+BSX+CTX	2 (2)				1 (2)	1 (3)	
No immunosuppression	1 (1)				1 (2)	0 (0)	
Tacrolimus blood levels at 1M, ng/mL (IQR)	10 (8 - 11)	N/A	N/A	N/A	9 (7-11)	10 (8-12)	0.264
Prophylaxis treatment at 1M, n (%)				N/A			0.890
TMP-SMX+VGCV	85 (90)	N/A	N/A		50 (89)	35 (90)	
TMP-SMX+VGCV+Others	7 (7)				4 (7)	3 (8)	
TMP-SMX+ACV+Others	1 (1)				1 (2)	0 (0)	
TMP+VGCV	1 (1)				0 (0)	1 (2)	
No prophylaxis	1 (1)				1 (2)	0 (0)	
EBV-serology pre-transplantation, n (%)				0.559			0.309
Positive	48 (51)	25 (46)	23 (56)		26 (46)	22 (56)	
Negative	40 (42)	24 (44)	16 (39)		27 (48)	13 (33)	
Unknown	7 (7)	5 (9)	2 (5)		3 (5)	4 (10)	
CMV-serology pre-transplantation, n (%)				0.300			0.980
Positive	55 (58)	35 (65)	20 (49)		31 (55)	24 (62)	
Negative	35 (37)	17 (31)	18 (44)		22 (39)	13 (33)	
Unknown	5 (5)	2 (4)	3 (7)		3 (5)	2 (5)	

1M, 1 month post-transplantation; ACV, acyclovir; BSX, Basiliximab, CE, corticosteroids; CMV, Cytomegalovirus; EBV, Epstein-Barr virus; IQR, interquartile range; MMF, mycophenolate mofetil; N/A, not applicable; CTX, chemotherapy, SMX, sulfamethoxazole; TAC, tacrolimus; TMP, trimethoprim; VGCV, valganciclovir.

Patients were monitored for 1 year. Follow-up periods included a baseline study just before transplantation (PreTx) and five studies post-transplantation at 1, 3, 6, 9 and 12 months after the procedure (1M, 3M, 6M, 9M and 12M). Demographic and clinically relevant information was collected (**Table 1**). Immune status was assessed at each timepoint. We considered clinically relevant infections, rejection, liver autoimmunity and PTLD as primary outcomes.

The standard induction regimen consisted of basiliximab administered on days 0 and 4 post-transplantation, combined with tacrolimus and corticosteroids. Maintenance immunosuppression mainly consisted of tacrolimus and corticosteroids. In selected cases experiencing rejection episodes, mycophenolate mofetil was added to the regimen.

Antimicrobial prophylaxis included trimethoprim-sulfamethoxazole for Pneumocystis jirovecii, administered for two years post-transplantation, and either ganciclovir or valganciclovir for Cytomegalovirus, prescribed for six months post-transplantation regardless of donor/recipient serostatus.

Infectious events were categorized according to Van Delden et al. (20), and their relevance was defined as proven bacterial, probable/proven fungal and probable/proven viral infections, as well as viral syndromes. Early infections were defined as those occurring within the first month post-transplantation, while infections occurring thereafter were classified as late infections. Autoimmune hepatitis (AIH) was defined by a positive test result for any of the following antibodies: anti-mitochondrial M2, anti-filamentous-actin (F-actin), anti-Liver Cytosol Antigen Type 1 or anti-Liver-Kidney Microsomal antibodies, along with meeting clinical criteria. PTLD diagnosis was based on histopathologic criteria. The histopathological diagnosis of acute allograft rejection was determined based on the Banff criteria (21).

### 2.2 Immune status assessment

Cellular immune status was evaluated by multiparametric flow cytometry. Briefly, 75µL of whole blood was stained with various monoclonal antibody combinations, using different panels over time due to supplier changes (**Supplementary Table S1**). Comparative analyses were conducted to ensure that the percentages remained consistent across all panels (data not shown). Cell acquisition was made on a BD FACSCanto™ or a DxFLEX flow cytometer. The resulting data were analyzed by FACSDiva™ (BD, USA) or Kaluza (Beckman Coulter, USA) software.

Immunophenotype of T lymphocytes (CD3^+^, further classified as CD4^+^ and CD8^+^), B lymphocytes (CD19^+^), NK lymphocytes (CD3^-^CD16^+^CD56^+^) and NKT cells (CD3^+^CD16^+^CD56^+^) was performed. CD4^+^ and CD8^+^ T lymphocytes were further distributed in naïve (Tn, CD27^+^CD45RO^-^), effector (Teff, CD27^-^CD45RO^-^), central memory (Tcm, CD27^+^CD45RO^+-^) and effector memory (Tefm, CD27^-^CD45RO^+-^) subsets. Additional quantified subpopulations included recent thymic emigrants (RTE, CD4^+^CD45RA^+^CD31^+^), Treg (CD3^+^CD4^+^CD25^+^CD127^low^), gamma-delta T lymphocytes (Tγδ, CD3^+^TCRγδ^+^), activated T cells (CD3^+^HLA-DR^+^) and memory B cells (Bm, CD19^+^CD27^+^).

Absolut numbers of T, B and NK lymphocytes were normalized to a patient-specific age range (22), creating a variable called “score”. To calculate the score, the median of the age-specific normal range was subtracted from the absolute number of lymphocytes in the subpopulation. The result was then divided by the difference between the 90^th^ percentile and the 10^th^ percentile of the normal range for that age group. Lymphopenia was defined as a score under -0.5 and lymphocytosis as a score over 0.5.

Regarding humoral immunity, levels of immunoglobulins G, A and M (IgG, IgA and IgM) were quantified on serum by nephelometry following manufacturer’s instructions (Siemens, Altona). Hypogammaglobulinemia was defined as values of IgG, IgA or IgM below the lower 95% confidence interval for each age group (23).

### 2.3 Statistical analysis

Quantitative variables were compared between two groups using the Mann–Whitney U test, except for the Δscore, for which the Student’s t-test was applied after confirming normal distribution with the Shapiro–Wilk test. When comparing quantitative variables across more than two groups, the Kruskal-Wallis test was used, followed by Dunn’s *post hoc* test for pairwise comparisons. Survival analysis was performed using the Cox proportional hazards model. The optimal multivariate model was selected using the Akaike Information Criterion, starting with variables with a p-value <0.100 from univariate analysis. The final model retained variables with the best fit. Statistical significance was set at p <0.05. All analyses were conducted with RStudio (version 4.3.3, R Core Team, 2024).

## 3 Results

### 3.1 Baseline clinical features

Ninety-five patients were ultimately included in our prospective study (**Figure 1**), with a median age of 16 (7–88) months. The baseline characteristics of the cohort are detailed in **Table 1**. Split graft from a deceased donor was the most common type of donation (41%), with biliary atresia being the predominant indication for LT (63%). Only 6 patients (6%) underwent combined liver-kidney transplantation, while 9 others (9%) required a second transplant due to primary graft failure (n=7), acute rejection (n=1) or tumor recurrence (n=1).

Ninety-seven percent of the patients received the standard induction regimen. Two patients (2%) also received mycophenolate as part of their induction therapy due to a combined transplant with a kidney graft, whereas one patient (1%) with a liver tumor was on chemotherapy at the time of transplantation.

Seven patients (7%) received intravenous immunoglobulin (IVIG). Two (2%) were prescribed IVIG prior to transplantation: one as part of the treatment for Gestational Alloimmune Liver Disease and another in the context of Evans syndrome associated with Autoimmune Lymphoproliferative Syndrome. Two patients (2%) received IVIG post-transplantation for the management of either adenovirus or Epstein–Barr virus infections. Three additional patients (3%) were treated with IVIG due to severe post-transplant hypogammaglobulinemia. Only two of these seven patients (29%) remained free of infections.

### 3.2 Events of rejection, AIH, PTLD and infection post-transplantation

Regarding post-transplant outcomes, 6 episodes of acute cellular rejection were diagnosed (6%) along the follow-up (median time 233 [50 – 349] days). Three patients had a diagnosis of AIH before transplantation: one with type 1 AIH, one with seronegative AIH and one with suspected AIH. One patient tested positive for anti-F-actin antibodies at a titer of 1:80 at 6M, though the antibody was undetectable in subsequent tests. Other autoimmune complications included one case of autoimmune neutropenia and one of autoimmune hemolytic anemia. PTLD was diagnosed in 2 patients (2%) at 6M and 9M, respectively. The low number of rejection, AIH or PTLD events reported prevented us from doing statistical analysis.

**Figure 2:**
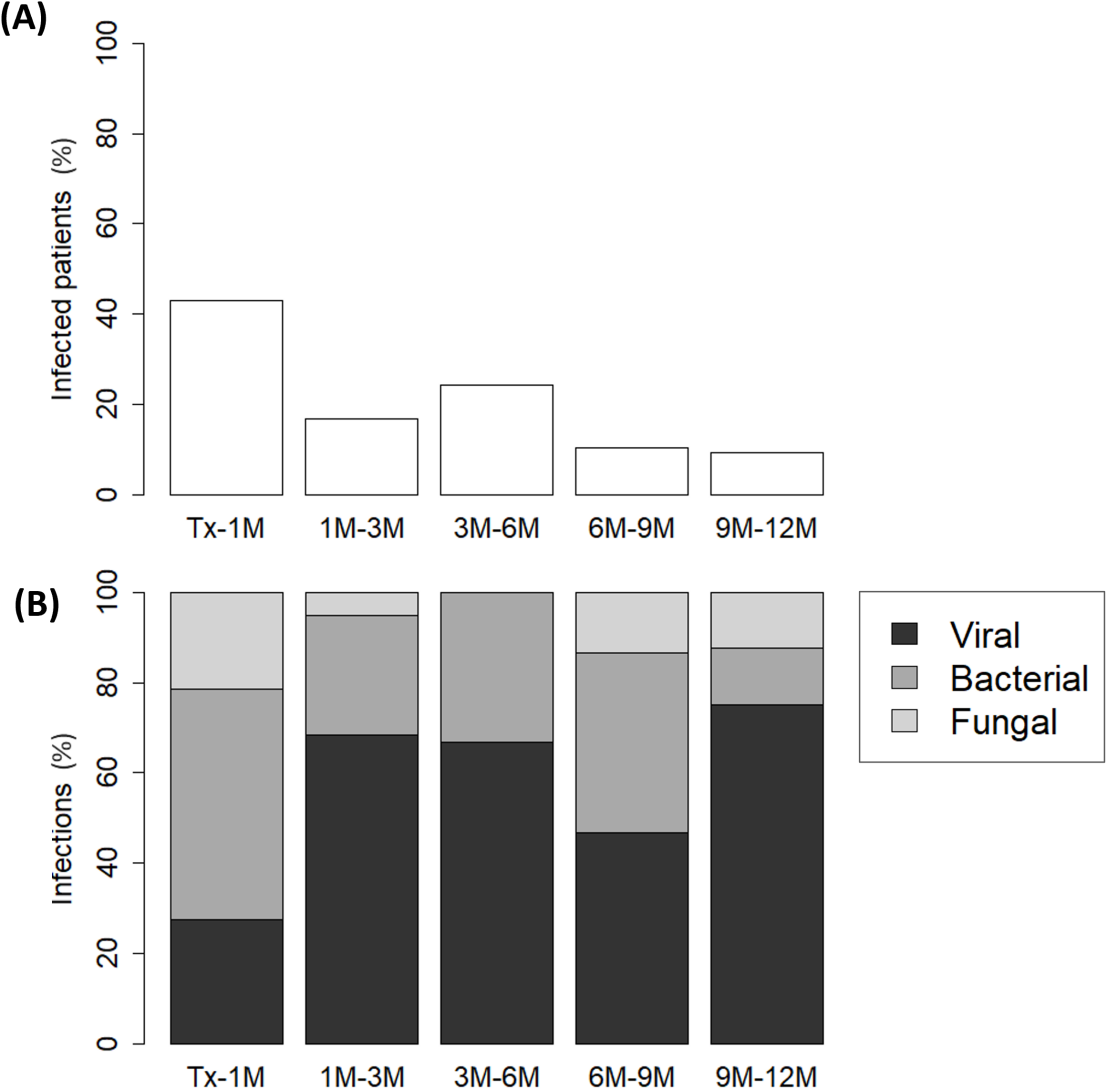
Percentage of **(A)** infected pediatric liver recipients and **(B)** distribution of infection types (viral, bacterial, or fungal) across each follow-up period: from transplantation (Tx) to 1 month post-transplantation (1M), 1M to 3M, 3M to 6M, 6M to 9M and 9M to 12M.

Regarding infections, most of them occurred within the first month post-transplantation (early infections) (median time 4 [1-12] days), affecting 41 patients (43%) (**Figure 2A**). In subsequent months, the proportion decreased to 17%, 24%, 10% and 9% during their respective follow-up periods (**Figure 2A**). Early infections were predominantly bacterial, accounting for 51% of cases (**Figure 2B**). In contrast, late infections (median time 100 [30-150] days) were primarily viral, comprising 68%, 67%, 47% and 75% of infections during the corresponding follow-up periods (**Figure 2B**). Pathogens causing early and late infections are detailed in **Supplementary Table S2**.

### 3.3 Evolution of immunoglobulins and lymphocyte populations during the first year post-transplantation

**Figure 3:**
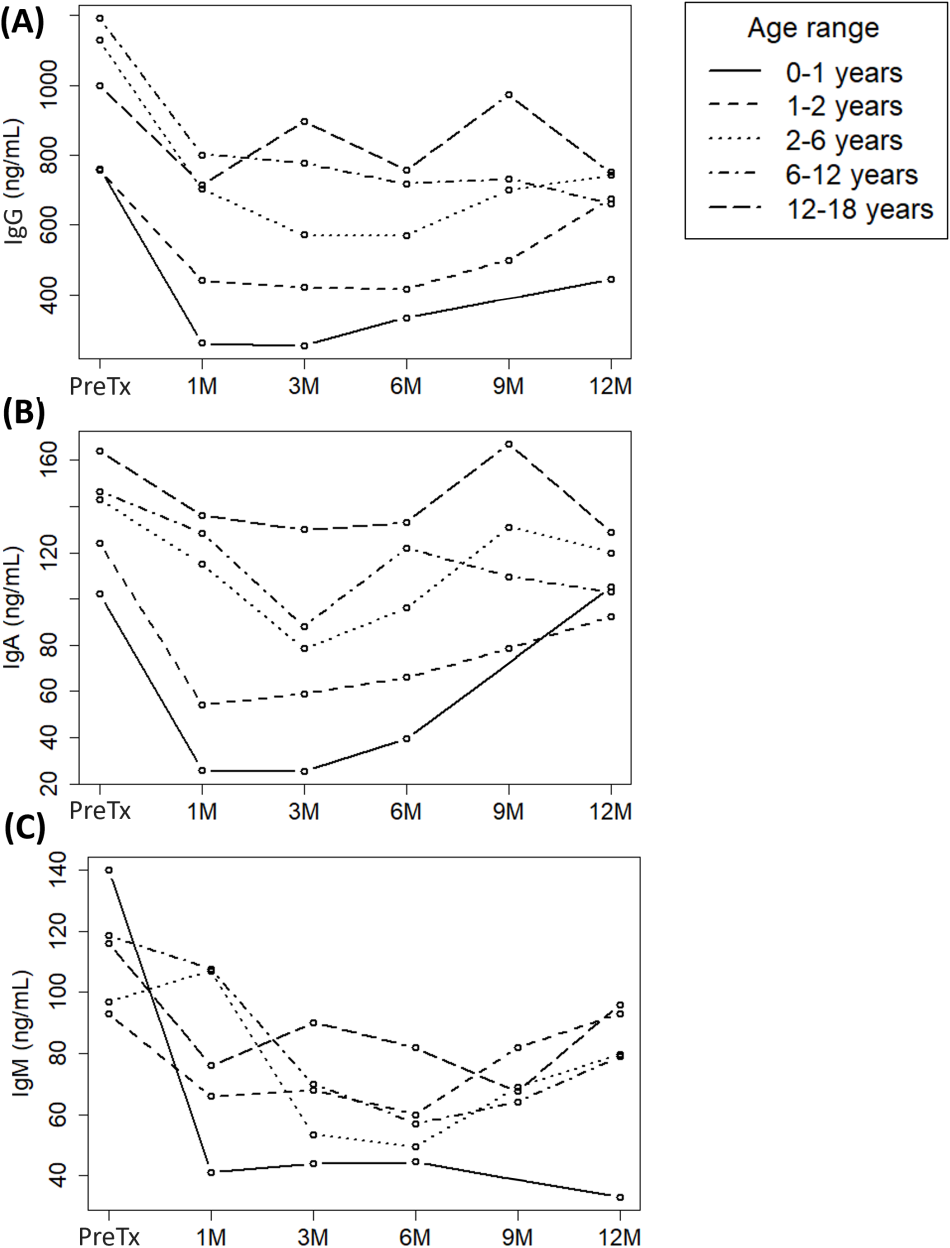
Evolution of **(A)** immunoglobulin G (IgG), **(B)** IgA and **(C)** IgM serum levels in a cohort of pediatric liver recipients grouped by age ranges across each follow-up period: pre-transplantation (Pre-Tx) and 1, 3, 6, 9 and 12 months post-transplantation (1M, 3M, 6M, 9M and 12M, respectively).

IgA hypogammaglobulinemia was detected in 2 (3%) recipients before the procedure, while the rest of patients maintained normal levels of both IgG and IgM (**Supplementary Table S3**). Transplantation had a negative impact on immunoglobulin levels during the first month post-transplantation, with 18 (20%), 19 (21%) and 17 (19%) recipients developing hypogammaglobulinemia for IgG, IgA, and IgM, respectively. During the subsequent months, immunoglobulin levels gradually increased (**Figures 3A-C**) and, by the end of the follow-up period, most patients had returned to normal levels. However, 7 (11%) patients still had IgM hypogammaglobulinemia, and 2 (3%) patients had hypogammaglobulinemia of either IgG or IgA (**Supplementary Table S3**).

**Figure 4:**
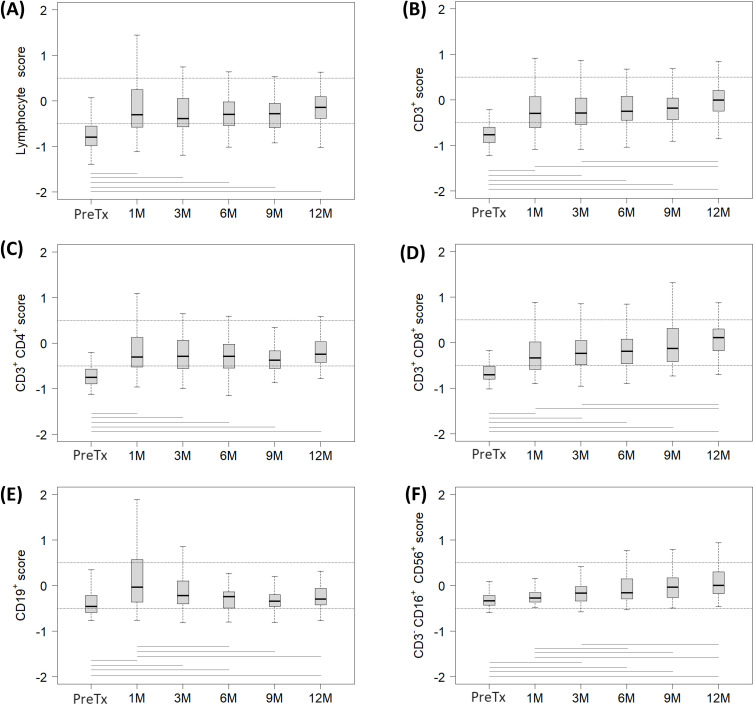
Evolution of each lymphocyte subpopulation score in a cohort of pediatric liver recipients across each follow-up period: pre-transplantation (Pre-Tx) and 1, 3, 6, 9 and 12 months post-transplantation (1M, 3M, 6M, 9M and 12M, respectively). Studied subsets included **(A)** total lymphocytes, **(B)** CD3^+^ T lymphocytes, **(C)** CD3^+^CD4^+^ T lymphocytes, **(D)** CD3^+^CD8^+^ T lymphocytes, **(E)** CD19^+^ B lymphocytes and **(F)** CD3^-^CD16^+^CD56^+^ NK lymphocytes. Scores were calculated by substracting the median of the age-specific normal range from the absolute number of lymphocytes in the subpopulation. Dashed lines mark the normal range, defined as scores between -0.5 and 0.5. Horizontal lines represent statistically significant differences between the median scores of two distinct follow-up periods.

Lymphopenia was frequent among recipients prior to transplantation (78%) (**Supplementary Table S3**), and mainly attributable to baseline CD3^+^ T lymphopenia (81%). In contrast, pre-transplant B and NK lymphopenia was observed in lower percentages (42% and 6%, respectively) (**Supplementary Table S3**). Accordingly, the baseline median score of total lymphocytes (**Figure 4A**) and T lymphocytes (**Figure 4B**) mirrored each other, both being below -0.5 prior to transplantation, including CD4^+^ (**Figure 4C**) and CD8^+^ (**Figure 4D**) T subsets. Upon transplantation, T lymphocytes already increased above -0.5 at 1M (**Figure 4B**), rising from -0.77 (-0.94 to -0.60) to -0.30 (-0.60 to 0.07) (p<0.001). Concomitantly, both CD4^+^ (PreTx -0.76 [-0.89 to -0.57] vs 1M -0.30 [-0.53 to 0.13], p<0.001) and CD8^+^ (PreTx -0.71 [-0.80 to -0.51] vs 1M -0.34 [-0.58 to 0.02], p<0.001) T-cell scores also exceeded -0.5 at 1M (**Figures 4C, D**, respectively).

CD3^+^ T lymphocytes at 1M and 3M were significantly lower compared to 12M values (1M -0.30 [-0.60 to 0.07] and 3M -0.30 [-0.54 to 0.04] vs 12M -0.01 [-0.24 to 0.20], p=0.005 and p=0.008, respectively), with the CD3^+^ T-cell score remaining comparable from 6M onwards. Regarding CD4^+^ T-cell score, it normalized at 1M and remained stable throughout the follow-up (**Figure 4C**), with no significant differences observed. Kinetics of the CD8^+^ T-cell score paralleled those described for CD3^+^ T lymphocytes (**Figure 4D**). CD8^+^ T lymphocytes at 1M and 3M were significantly lower compared to 12M values (1M -0.34 [-0.58 to 0.02] and 3M -0.24 [-0.49 to 0.05] vs 12M 0.11 [-0.18 to 0.30], p<0.001 and p=0.003, respectively). Normalization was observed at 6M, after which the CD8^+^ T-cell score remained stable.

Regarding B lymphocytes, a significant expansion at 1M was observed (PreTx -0.46 [-0.59 to -0.21] vs 1M -0.04 [-0.36 to 0.57], p<0.001) (**Figure 4E**), showing values over 0.5 score (lymphocytosis) in 27 (30%) patients at that timepoint. B-cell expansion observed at 1M significantly decreased by 6M (-0.25 [-0.49 to -0.14], p<0.001), when normalization was achieved. From that time onwards, B-cell score remained stable, with no significant differences observed.

The impact of transplantation on NK-cell score appeared less pronounced initially (**Figure 4F**). Compared to pre-transplant study, a significant increase was detected at 3M (PreTx -0.33 [-0.43 to -0.21] vs 3M -0.17 [-0.34 to -0.02], p<0.001). After 6M, NK-cell scores normalized and remained stable in the following months.

### 3.4 Evolution of expanded-phenotype cell populations during the first year post-transplantation

Firstly, the decrease in Treg subpopulation at 1M (**Table 2**) was likely an artifact of the technique, as basiliximab (anti-CD25) used in induction therapy interfered with CD25 detection by flow cytometry. Therefore, 1-month Treg frequencies were excluded from our analysis.

**Table 2:** Lymphocyte frequencies along the different follow-up periods in a cohort of pediatric liver recipients segregated by age ranges.

T-lymphocyte subset	PreTx		1M		3M		6M		9M		12M		p-value
	n	median (IQR)	n	median (IQR)	n	median (IQR)	n	median (IQR)	n	median (IQR)	n	median (IQR)	
Tγδ													
**0–1 years**	26	2.63 (1.68 - 3.58)^a,b^	28	3.22 (1.61 - 3.85)^c,d^	20	5.10 (3.49 - 8.67)^a,c^	6	9.01 (7.13 - 15.11)^b,d^	0	NA	1	22.29 (22.29 - 22.29)	<0.001
**1–2 years**	13	5.40 (4.35 - 5.82)^a,b^	19	3.48 (2.31 - 4.85)^c,d,e^	21	4.60 (2.93 - 6.10)^f,g^	33	6.37 (3.83 - 11.75)^c^	28	9.72 (7.56 - 14.21)^a,d,f^	24	10.62 (9.04 - 13.30)^b,e,g^	<0.001
**2–6 years**	15	6.16 (3.78 - 7.89)	17	3.90 (2.69 - 4.98)^a,b,c^	18	5.21 (3.10 - 8.92)^d^	16	8.09 (5.68 - 10.98)^a^	17	10.05 (7.43 - 13.80)^b^	20	11.81 (7.21 - 14.70)^c,d^	<0.001
**6–12 years**	12	8.67 (5.60 - 16.50)	12	6.66 (3.48 - 10.96)^a^	13	6.62 (5.67 - 12.52)^b^	13	11.14 (7.72 - 15.12)	12	15.54 (14.00 - 18.48)	11	17.56 (12.08 - 19.63)^a,b^	0.002
**12–18 years**	13	9.10 (5.64 - 11.40)	13	4.49 (3.60 - 8.12)	13	6.42 (4.53 - 9.58)	14	7.74 (5.11 - 11.97)	10	7.98 (7.44 - 12.69)	8	9.09 (6.97 - 15.10)	0.220
NKT													
**0–1 years**	26	0.26 (0.10 - 0.55)	28	0.29 (0.16 - 0.60)	20	0.29 (0.16 - 0.52)	6	1.03 (0.29 - 1.45)	0	NA	1	0.95 (0.95 - 0.95)	0.269
**1–2 years**	13	0.31 (0.21 - 0.99)	19	0.25 (0.19 - 0.36)^a,b^	21	0.49 (0.20 - 0.81)	33	0.46 (0.22 - 0.63)	28	0.61 (0.30 - 1.61)^a^	24	0.62 (0.32 - 1.15)^b^	0.006
**2–6 years**	15	0.41 (0.29 - 1.21)	17	0.38 (0.24 - 0.80)	18	0.50 (0.23 - 0.77)	16	0.65 (0.36 - 1.48)	17	0.56 (0.46 - 0.80)	20	0.88 (0.53 - 1.39)	0.111
**6–12 years**	12	1.20 (0.67 - 2.33)	12	0.95 (0.62 - 1.93)	13	0.86 (0.66 - 1.13)	13	1.39 (1.10 - 2.78)	12	1.05 (0.72 - 1.58)	11	1.26 (1.11 - 1.97)	0.388
**12–18 years**	13	3.40 (1.10 - 4.15)	13	0.87 (0.60 - 2.16)	13	1.14 (0.80 - 3.02)	14	1.68 (1.07 - 4.23)	10	2.63 (1.54 - 4.28)	8	2.49 (1.49 - 4.92)	0.234
Treg													
**0–1 years**	26	8.95 (5.07 - 11.30)	25	0.70 (0.02 - 7.00)*	20	8.33 (6.82 - 10.39)	5	8.81 (8.06 - 10.24)	0	NA	1	3.49 (3.49 - 3.49)	0.113
**1–2 years**	13	8.95 (5.07 - 11.30)	18	0.00 (0.00 - 0.30)*	18	8.49 (7.63 - 9.58)^a,b^	29	6.46 (5.62 - 7.53)	26	5.10 (4.04 - 6.76)^a^	24	5.17 (3.96 - 6.11)^b^	<0.001
**2–6 years**	15	8.62 (4.95 - 11.05)	16	5.82 (1.07 - 11.59)*	18	7.72 (6.38 - 10.66)	15	8.34 (6.04 - 9.01)	17	6.02 (5.01 - 7.02)	20	5.55 (4.98 - 7.31)	0.069
**6–12 years**	12	6.60 (5.46 - 8.57)	11	0.45 (0.00 - 4.92)*	13	7.49 (6.90 - 13.70)	13	5.80 (4.72 - 8.79)	12	5.12 (4.38 - 7.85)	11	6.55 (4.56 - 7.96)	0.198
**12–18 years**	13	6.66 (4.52 - 8.34)	12	0.02 (0.00 - 1.66)*	13	6.56 (4.96 - 7.73)	11	6.74 (5.39 - 7.14)	10	5.75 (4.59 - 6.68)	8	4.83 (4.58 - 6.31)	0.621
RTE													
**0–1 years**	26	64.48 (56.75 - 75.83)	27	61.60 (56.61 - 72.32)	19	53.52 (47.34 - 67.34)	6	64.20 (44.83 - 69.66)	0	NA	1	46.51 (46.51 - 46.51)	0.278
**1–2 years**	13	56.16 (45.15 - 66.04)	19	67.20 (57.24 - 74.30)^a,b^	20	57.36 (45.12 - 71.54)	31	52.07 (39.15 - 60.62)^a^	28	52.79 (42.76 - 59.54)^b^	24	52.23 (46.63 - 58.75)	0.020
**2–6 years**	14	44.86 (36.89 - 48.80)	17	51.75 (37.17 - 58.72)	18	47.38 (41.94 - 58.76)	16	48.12 (38.49 - 56.53)	17	44.74 (35.30 - 62.22)	20	46.22 (32.38 - 51.52)	0.654
**6–12 years**	12	46.78 (40.36 - 53.59)	12	46.67 (36.46 - 53.26)	12	52.98 (45.17 - 55.59)	13	48.40 (43.10 - 54.23)	12	47.31 (45.19 - 50.66)	11	38.92 (38.28 - 51.35)	0.693
**12–18 years**	13	44.08 (37.40 - 48.31)	13	50.20 (36.30 - 54.22)	13	47.46 (34.21 - 52.41)	14	47.74 (32.57 - 49.90)	10	43.67 (29.34 - 45.56)	8	42.41 (30.70 - 52.22)	0.687
Bm													
**0–1 years**	26	8.98 (5.93 - 14.75)	28	7.29 (4.29 - 11.73)	20	7.08 (5.59 - 8.46)	6	11.73 (8.06 - 13.97)	0	NA	1	8.20 (8.20 - 8.20)	0.386
**1–2 years**	13	8.60 (6.62 - 15.80)	19	9.33 (6.70 - 11.74)	21	9.24 (7.83 - 12.55)	33	11.04 (7.64 - 15.13)	28	12.15 (9.62 - 16.36)	24	11.92 (8.68 - 15.88)	0.446
**2–6 years**	15	10.67 (7.43 - 14.02)^a^	17	11.74 (8.01 - 14.50)^b^	18	13.21 (10.81 - 16.74)	16	17.32 (14.10 - 21.13)	17	16.60 (13.15 - 20.96)	20	20.33 (15.68 - 22.53)^a,b^	0.003
**6–12 years**	12	15.80 (11.82 - 23.05)	11	14.29 (10.79 - 16.34)	13	13.77 (9.80 - 17.93)	13	17.52 (10.28 - 22.57)	12	14.17 (10.36 - 26.72)	10	12.97 (11.46 - 14.31)	0.911
**12–18 years**	13	20.10 (8.10 - 24.85)	13	18.50 (5.36 - 22.62)	13	16.61 (10.05 - 28.00)	14	11.53 (7.53 - 19.01)	10	14.45 (8.83 - 17.17)	8	15.64 (8.02 - 20.91)	0.961
CD3^+^HLA-DR^+^													
**0–1 years**	26	6.45 (3.69 - 17.21)	28	4.92 (3.58 - 11.15)	20	8.80 (4.90 - 11.88)	6	17.57 (9.04 - 18.23)	0	NA	1	8.41 (8.41 - 8.41)	0.246
**1–2 years**	13	17.80 (10.94 - 21.32)	19	7.51 (3.72 - 12.79)^a^	21	13.26 (8.60 - 19.61)	33	15.48 (11.16 - 22.08)	28	17.79 (10.39 - 29.34)^a^	24	21.18 (9.59 - 28.73)	0.040
**2–6 years**	15	14.55 (9.04 - 29.80)	17	10.00 (5.08 - 12.11)^a^	18	7.26 (5.56 - 17.03)^b^	16	8.24 (6.09 - 12.35)^c^	17	16.95 (7.27 - 34.11)	20	26.16 (17.29 - 30.41)^a,b,c^	0.001
**6–12 years**	12	10.40 (6.68 - 14.67)	12	13.23 (9.31 - 20.36)	13	18.16 (4.69 - 22.10)	13	13.55 (7.37 - 30.90)	12	11.82 (7.04 - 20.43)	11	26.56 (12.44 - 35.21)	0.368
**12–18 years**	13	15.40 (10.48 - 20.30)	13	14.70 (8.70 - 23.33)	13	13.50 (12.20 - 19.37)	14	22.31 (12.46 - 30.75)	10	32.92 (19.08 - 37.09)	8	21.28 (15.32 - 35.97)	0.248

1M, 1 month post-transplantation; 3M, 3 months post-transplantation; 6M, 6 months post-transplantation; 9M, 9 months post-transplantation; 12M, 12 months post-transplantation; Bm, memory B lymphocytes; CD3+HLA-DR+, activated T lymphocytes; IQR, interquartile range; PreTx, pre-transplantation; NA, not applicable; RTE, recent thymic emigrants lymphocytes; Treg, regulatory T lymphocytes; Tγδ, gamma-delta T lymphocytes.

^a-g^Significant differences (p<0.05).

*Frequencies were ommited from the analysis due to the impossibility to detect CD25 by flow cytometry for some patients.

Infant patients aged 0–1 year (**Table 2**) showed a significant increase in Tγδ lymphocyte frequency, rising from 2.63% pre-transplantation and 3.22% at 1M to 5.10% at 3M (p= 0.008 and p =0.001, respectively) and 9.01% at 6M (p=0.015 and p=0.002, respectively). In recipients aged 1–2 years, Tγδ lymphocyte frequency significantly increased from 5.40% pre-transplantation to 9.72% at 9M and 10.62% at 12M (p=0.005 and p=0.003, respectively). This rise was also significant when comparing 1M (3.48%) to 6M (6.37%), 9M and 12M (p=0.008, p<0.001 and p<0.001, respectively), and when comparing 3M (4.60%) to 9M and 12M (p<0.001 for both comparisons).

Regarding NKT lymphocytes in patients aged 1–2 years (**Table 2**), their frequency increased from 0.25% at 1M to 0.61% at 9M (p= 0.003) and 0.62% at 12M (p=0.006). Conversely, Treg frequency decreased from 8.49% at 3M to 5.10% at 9M (p<0.001) and 5.17% at 12M (p<0.001), while the frequency of RTE declined from 67.20% at 1M to 52.07% at 6M and 52.79% at 9M (p= 0.015 and p= 0.012, respectively). In contrast, activated CD3^+^HLA-DR^+^ T lymphocytes showed an increase from 7.51% at 1M to 17.79% at 9M (p=0.023).

Similarly, patients aged 2–6 years increased their Tγδ and CD3^+^HLA-DR^+^ subsets throughout the follow-up period (**Table 2**). Interestingly, frequencies of Bm only showed an increase in that age group, rising from baseline 10.67% and 11.74% at 1M to 20.33% at 12M (p=0.007 and p=0.016, respectively). On the other hand, in older patients aged 6–12 years, only an increase in Tγδ lymphocytes from 6.66% at 1M and 6.62% at 3M to 17.56% at 12M (p=0.009 and p=0.008, respectively) was detected. For recipients aged 12–18 years, the frequencies remained stable throughout the entire follow-up period, with multiple comparisons yielding no significant p-values.

The distribution by age of CD4^+^ and CD8^+^ Tn, Teff, Tcm, and Tefm lymphocyte subpopulations throughout the follow-up period remained comparable (**Supplementary Figures S1**, **S2**). However, in patients aged from 2–6 years, median frequencies of CD8^+^ Tn significantly decreased from 1M to 12M (75.61% vs 54.20%, p=0.012). Conversely, CD8^+^ Teff and Tefm subsets in this age group significantly increased in the same period (CD8^+^ Teff 1.71% vs 11.34%, p=0.005; CD8^+^ Tefm 3.81% vs 10.00%, p=0.010) (**Supplementary Figure S2**).

### 3.5 Association of T-cell lymphopenia and hypogammaglobulinemia with the risk of infection

When segregated according to the time of infection (early/late), statistical analysis showed that infected patients were significantly younger at transplant and primarily received split grafts (**Table 1**). To better assess immunological parameters post-transplantation, we subtracted each subpopulation score from pre-transplant study to the one obtained at 1M (Δscore). The higher the Δscore, the better the normalization of lymphocyte subpopulations. Patients that remained free from late infections had higher Δscore for T CD3^+^ (0.524 vs 0.263, p=0.018) and T CD4^+^ (0.452 vs 0.287, p=0.036) than those who developed late infections (**Table 3**). Interestingly, T CD8^+^ Δscore was also higher in non-infected patients, although this increase nearly reached statistical significance (0.483 vs 0.178, p=0.054).

**Table 3:** Estimation of cellular immunity recovery following pediatric liver transplantation calculated by differences between pre-transplant score and 1-month post-transplant score (Δscore).

Δscore	Infection	No infection	p-value
	(n=35)	(n=41)	
**Lymphocytes**	0.342 (0.017 to 0.960)	0.532 (0.172 to 0.961)	0.155
**T cell CD3^+^**	0.263 (-0.071 to 0.680)	0.524 (0.227 to 0.906)	0.018
**T cell CD3^+^CD4^+^**	0.287 (0.001 to 0.695)	0.452 (0.244 to 0.882)	0.036
**T cell CD3^+^CD8^+^**	0.178 (-0.058 to 0.666)	0.483 (0.207 to 0.753)	0.054
**B cell CD19^+^**	0.280 (0.070 to 1.065)	0.392 (0.130 to 1.025)	0.679
**NK cell CD3^-^CD16^+^ CD56^+^**	0.049 (-0.168 to 0.253)	0.088 (-0.053 to 0.208)	0.767

Subsequently, a survival analysis was performed to explore the relationship between pre-transplant immunological status and the risk of early infections (**Table 4**). The univariate analysis identified a significant association between the risk of post-transplant infections and both the age at transplantation and the type of graft. Patients aged 0–1 years (hazard ratio [HR] 5.23, p=0.027) or 1–2 years (HR 5.29, p=0.034) had a significantly higher risk of infection. Transplantation using a split graft was associated with a threefold risk for infection (HR 3.02, p=0.071), although this correlation was not statistically significant in the univariate analysis. None of the immunological variables analyzed were associated with the risk of early infection. Interestingly, in the multivariate analysis, only transplantation with a split graft was independently associated with an increased risk of early infection (HR 3.42, p=0.047).

**Table 4:** Early infection univariate and multivariate analysis in a cohort of pediatric liver recipients categorized by their immune status of lymphopenia or hypogammaglobulinemia pre-transplantation.

Baseline characteristics	n	UNIVARIATE		MULTIVARIATE	
		HR (95% CI)	p-value	HR (95% CI)	p-value
Age (years)					
0-1	31	5.23 (1.21 - 22.58)	0.027	3.09 (0.58 – 16.30)	0.185
1-2	16	5.29 (1.14 – 24.57)	0.034	3.78 (0.70 – 20.43)	0.122
2-6	18	3.82 (0.82 – 17.84)	0.089	2.15 (0.41 – 11.36)	0.370
6-12	15	2.13 (0.39 – 11.61)	0.384	1.33 (0.23 – 7.61)	0.750
12-18	15	Reference		Reference	
Type of donor					
Deceased donor - reduced graft	23	2.14 (0.60 – 7.60)	0.242	3.75 (0.96 – 14.73)	0.058
Deceased donor - split graft	39	3.02 (0.91 – 10.04)	0.071	3.42 (1.02 – 11.50)	0.047
Deceased donor - whole graft	22	0.48 (0.10 - 2.39)	0.371	1.00 (0.18 – 5.67)	0.997
Living donor	11	Reference		Reference	
Lymphopenia					
Yes	63	2.38 (0.84 – 6.74)	0.102		
No	16	Reference			
Lymphopenia T CD3^+^					
Yes	64	2.16 (0.77 – 6.13)	0.145		
No	15	Reference			
Lymphopenia T CD3^+^CD4^+^					
Yes	64	1.60 (0.62 – 4.12)	0.330		
No	15	Reference			
Lymphopenia T CD3^+^CD8^+^					
Yes	60	1.93 (0.75 – 4.97)	0.173		
No	19	Reference			
Lymphopenia B CD19^+^					
Yes	63	1.19 (0.62 – 2.29)	0.603		
No	16	Reference			
Lymphopenia NK CD3^-^CD16^+^CD56^+^					
Yes	5	0.82 (0.20 – 3.41)	0.783		
No	74	Reference			
Hypogammaglobulinemia IgG					
Yes	0	NA	NA		
No	76	Reference			
Hypogammaglobulinemia IgA					
Yes	2	1.58 (0.23 – 11.56)	0.652		
No	74	Reference			
Hypogammaglobulinemia IgM					
Yes	0	NA	NA		
No	76	Reference			

CI, confidence interval; Ig, immunoglobulin; NA, not applicable; NK, natural killer; OR, odds ratio.

**Figure 5:**
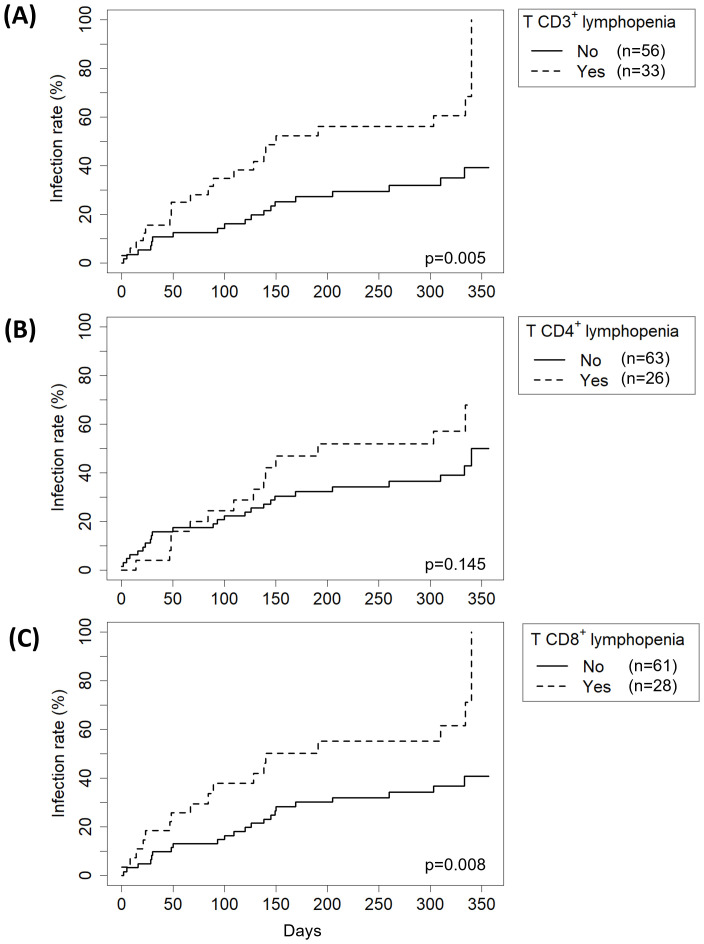
Effect of the presence of **(A)** T CD3^+^ T lymphopenia, **(B)** CD4^+^ T lymphopenia or **(C)** CD8^+^ T lymphopenia on late infection risk. Global p-values were obtained at 1 month post-transplantation by Kaplan-Meier analysis.

We next analyzed how immune status at 1M influenced the likelihood of remaining free from late infection (**Table 5**). The univariate model revealed that infants aged 0–1 years had a significantly higher risk of late infection (HR 3.49, p=0.046). Conversely, patients who received a whole graft from deceased donor had a significantly lower risk (HR 0.28, p=0.044). In terms of immunological status, CD3^+^ or CD8^+^ T lymphopenia (HR 2.48, p=0.005 and HR 2.38, p=0.008, respectively) and hypogammaglobulinemia (IgG, IgA or IgM), were associated with a higher risk of late infection (HR 2.18, p=0.036 and HR 2.40, p=0.011 and HR 2.61, p=0.006, respectively). The multivariate model showed that only lymphopenia T CD3^+^ was independently associated with an increased risk of late infection (HR 2.13, p=0.030). Kaplan-Meier curves for patients with or without T lymphopenia are graphed in **Figure 5**. Patients with CD3^+^ T lymphopenia showed significantly higher infection rates after the first month post-transplantation (p=0.005) (**Figure 5A**). While CD4^+^ T lymphopenia did not show a statistically significant association with infection rates (**Figure 5B**), the presence of CD8^+^ T lymphopenia was significantly associated with higher infection rates, highlighting the differential impact of T-cell subsets on infection risk (**Figure 5C**).

**Table 5:** Late infection univariate and multivariate analysis in a cohort of pediatric liver recipients categorized by their immune status of lymphopenia or hypogammaglobulinemia at one month post-transplantation.

One month post-transplantation characteristics	n	UNIVARIATE		MULTIVARIATE	
		HR (95% CI)	p-value	HR (95% CI)	p-value
Age (years)					
0-1	28	3.49 (1.02 - 11.92)	0.046		
1-2	19	3.28 (0.92 - 11.65)	0.066		
2-6	17	1.47 (0.37 - 5.87)	0.589		
6-12	12	0.32 (0.03 - 3.10)	0.327		
12-18	13	Reference			
Type of donor					
Deceased donor - reduced graft	22	0.39 (0.13 - 1.16)	0.090	0.50 (0.16 - 1.20)	0.220
Deceased donor - split graft	39	1.09 (0.46 - 2.55)	0.852	1.17 (0.48 - 2.82)	0.730
Deceased donor - whole graft	17	0.28 (0.08 - 0.97)	0.044	0.30 (0.09 - 1.05)	0.060
Living donor	11	Reference		Reference	
Lymphopenia					
Yes	32	1.88 (0.99 - 3.55)	0.053		
No	57	Reference			
Lymphopenia T CD3^+^					
Yes	33	2.48 (1.32 - 4.67)	0.005	2.13 (1.08 - 4.21)	0.030
No	56	Reference		Reference	
Lymphopenia T CD3^+^CD4^+^					
Yes	23	1.64 (0.84 - 3.17)	0.145		
No	66	Reference			
Lymphopenia T CD3^+^CD8^+^					
Yes	28	2.38 (1.26 - 4.50)	0.008		
No	61	Reference			
Lymphopenia B CD19^+^					
Yes	10	0.77 (0.24 - 2.50)	0.664		
No	79	Reference			
Lymphopenia NK CD3^-^CD16^+^CD56^+^					
Yes	0	NA	NA		
No	89	Reference			
Hypogammaglobulinemia IgG					
Yes	17	2.18 (1.05 - 4.51)	0.036		
No	72	Reference			
Hypogammaglobulinemia IgA					
Yes	19	2.40 (1.22 - 4.72)	0.011		
No	70	Reference			
Hypogammaglobulinemia IgM					
Yes	17	2.61 (1.31 - 5.19)	0.006	1.90 (0.91 – 3.95)	0.087
No	78	Reference		Reference	

CI, confidence interval; HR, hazard ratio; Ig, immunoglobulin; NA, not applicable; NK, natural killer.

Furthermore, we stratified patients into four groups based on IgG levels and CD3^+^ T-cell counts at 1M: normal IgG/normal CD3^+^ (n=50), normal IgG/CD3^+^ T lymphopenia (n=22), IgG hypogammaglobulinemia/normal CD3^+^ (n=6) and IgG hypogammaglobulinemia/CD3^+^ T lymphopenia (n=11). The risk of late infection was significantly higher in patients with normal IgG/CD3^+^ T lymphopenia (HR 3.03, 95% CI 1.46–6.30, p=0.003), IgG hypogammaglobulinemia/normal CD3^+^ (HR 4.16, 95% CI 1.36–12.74, p=0.013) and IgG hypogammaglobulinemia/CD3^+^ T lymphopenia (HR 2.86, 95% CI 1.10–7.44, p=0.031), compared to the normal IgG/normal CD3^+^ group.

## 4 Discussion

This study aimed to define the immune changes in pediatric LT and identify markers related to post-transplant complications. Our findings revealed that baseline T lymphopenia and first-month post-transplant IgG hypogammaglobulinemia mostly recover early in the follow-up. Additionally, patients with T CD3^+^ lymphopenia at 1M have a twofold increased risk of late infections.

In our cohort, end-stage liver disease negatively impacted the cellular compartment. Although the detrimental effect of biliary atresia on cellular immunity has been previously described (24), we found no association when comparing baseline immune scores of patients grouped by their underlying diagnosis (**Supplementary Table S4**). However, in line with previous results published by Möhring et al. (25), patients with liver tumors had the highest numbers of lymphocytes when adjusted for age (median score -0.63 [-0.89 to -0.37]). In a cohort of 60 adult patients with cirrhosis T CD4^+^ lymphopenia was observed (26), attributing it to defective lymphocyte production, splenic pooling and apoptosis from bacterial translocation. This may help understanding the variations in immune pre-transplant status within our cohort.

LT differently affected humoral and cellular immunity. Humoral immunity was negatively affected by LT, since patients who did not have hypogammaglobulinemia prior to the transplant developed it after receiving the graft. Our results are consistent with previous findings in pediatric liver recipients (14, 27), and are likely due to the excessive loss of immunoglobulin-rich ascitic serum during surgery. However, the effect of the immunosuppressive treatment should also be considered.

Conversely, cellular immunity immediately benefits from LT, as the frequency of patients with total lymphopenia decreased from 78% PreTx to 36% at 1M. In a cohort of 304 adult kidney recipients, lymphocyte subpopulations were analyzed at PreTx, 1M and 6M. Consistent with our results, those without anti-thymocyte globulin induction showed increased T-cell counts at 1M (28).

Our approach focuses on simultaneously monitoring humoral and cellular immunity after LT, allowing us to determine the timepoint at which normalization occurs for each Ig and lymphocyte subpopulation. The number of patients with hypogammaglobulinemia of any isotype began to decrease immediately after 1M. By 12M, only two patients showed IgG hypogammaglobulinemia, both of whom had received rituximab treatment for either PTLD or autoimmune hemolytic anemia. While CD3^+^ and CD8^+^ T-cell score normalization occurred at 6M, CD4^+^ T-cell score normalized at 1M; on the other hand, B-cell and NK-cell scores normalized at 6M. Interestingly, we noticed a remarkable expansion of B cells at 1M, likely compensating for the hypogammaglobulinemia present at that time.

Regarding expanded-phenotype subpopulations, transplantation had a less pronounced impact, since differences with PreTx values were only found for Tγδ lymphocytes in patients aged 0–2 years and Bm lymphocytes in patients 2–6 years old. Further differences appeared at 9M and 12M, likely reflecting age-related changes occurring throughout the follow-up period. This is supported by the absence of significant differences in patients over 12 years, suggesting diminished age-related fluctuations as patients mature. However, stratifying patients by age resulted in a reduced sample size in each subgroup, which may have limited the statistical power to detect additional differences.

Similarly, slight differences appeared in frequencies of Tn, Teff, Tcm and Tefm subsets. As children age, the frequency of Tn cells decreases, while the percentage of Teff, Tefm, and Tcm subpopulations increase, as anticipated (29). However, in patients aged 2–6 years, there was a significant decrease in CD8^+^ Tn cells and a significant increase in CD8^+^ Teff and Tefm subsets from 1M onwards, which might be related with cytotoxic immune response to viral late infections at that period.

In our cohort, acute cellular rejection occurred in 6% of patients, lower than previously reported. A 2004 study of 1,092 pediatric LT found a 48.4% incidence, with biopsies confirming 92% of cases (30), while a recent study in 50 pediatric cases reported a 68% incidence, with biopsies conducted at the physician’s discretion (31). The absence of serial biopsies in our cohort may have led to an underestimation of the true incidence, as subacute rejections could have been missed.

Autoimmune and PTLD complications were rare in our cohort. None of the recipients developed either *de novo* or recurrent AIH, despite reported incidences in pediatric LT of 1-11% (onset at 2–12 years post-LT) (32) and 38-89% (onset at 11–43 months) (33), respectively. Similarly, PTLD was diagnosed in 2% of our recipients, lower than reported incidences of 7.8-9.7% (4, 12). This may be attributed to our relatively short 1-year follow-up period compared to the 4–12 years of follow-up in other studies (4, 12).

Consistent with previous reports, early infections in our cohort were associated with surgery, while late infections resulted from heightened immunosuppression (34, 35). Thus, bacterial infections dominated the first month, whereas opportunistic viral infections become more frequent thereafter, due to prolonged immunosuppressive therapy (34–37). Since most of the studies have focused on adult liver recipient (14–16, 38–40), we specifically monitored the immune status in pediatric recipients to better assess their risk of infections.

Previous research had established that pre-transplant lymphopenia increases infection risk in adult LT (39, 40). Furthermore, Lei et al. found an association between the number of pre-transplant double-negative CD3^+^CD4^-^CD8^-^ T-cells and infection risk in a cohort of 19 adult LT (38). However, we did not identify pre-transplant immunological predictors for early infections in pediatric patients. Instead, split graft recipients were at a higher risk of early infections, likely due to increased biliary leakage leading to severe infections (41).

In contrast, we found that T CD3^+^ lymphopenia at 1M was associated with increased risk of late infections. This is consistent with Fernandez-Ruiz et al., who observed that adult kidney-transplant recipients with T CD8^+^ lymphopenia had a threefold increased risk of late infections (28). Interestingly, although in our multivariate analysis we did not find an association with hypogammaglobulinemia, other prospective studies have reported that infected adult liver recipients had lower IgG levels at days 3 (15) or 7 (16) post-transplantation. In line with these findings, our stratified analysis revealed that both isolated and combined alterations in IgG levels and CD3^+^ T-cell counts at 1M were associated with a significantly increased risk of late infections.

Previous studies have shown that lymphopenia is associated with an increased risk of both opportunistic and community-acquired infections. A large Danish cohort study in the general population demonstrated that individuals with lymphopenia had a significantly higher risk of hospital admission with an infection, as well as infection-related mortality (42). Similarly, in patients with solid tumors, radiation-induced lymphopenia has been linked to an elevated risk of bacterial infections (43). These findings support the relevance of peripheral T-cell counts as general markers of immune competence and infection susceptibility.

Beyond the markers explored in this research, assessing immune function could provide additional insights. A prospective study by Sood et al. (n=75) demonstrated that low interferon-gamma production after non-pathogen specific stimulation at week 1 post-transplant was associated with a higher risk of early infections, whereas elevated levels correlated with an increased risk of rejection (44). Incorporating such functional assays alongside markers like CD64 monocyte counts (15) or PD1 exhaustion marker (38) may enhance our ability to predict infection risk.

To our knowledge, this is the first prospective study monitoring the immune response of pediatric liver recipients. The ChilSFree cohort study proposed a similar approach (8), but results are yet to be reported. Based on our findings, we propose that measuring serum Ig levels, T (including CD4^+^ and CD8^+^ subsets), B and NK lymphocytes at PreTx, 1M, 6M and 12M provides a comprehensive assessment of immune recovery and identifies late infections risks. To validate these results, future multicenter studies should adopt a standardized protocol across all participating centers. Sample collection timepoints and technical procedures must be harmonized, and inclusion criteria and clinical endpoints unified. Such collaborative efforts would not only confirm the utility of these biomarkers but also support the development of personalized immunosuppression strategies in pediatric liver transplantation.

A key limitation of our study is the low incidence of autoimmune complications, PTLD and rejection, which restricted our ability to identify additional markers. Moreover, the lack of a more detailed classification of infections based on anatomical site and clinical severity limits our ability to accurately differentiate community-acquired infections from those opportunistic infections. Another limitation is the lack of immune function analysis. Thus, further studies with larger cohorts and immune function assessment are necessary to better understand the immunological landscape of post-transplant complications.

In conclusion, we showed that pediatric liver recipients have baseline immune dysregulation that is resolved during the first months after transplantation. While early infections in our cohort did not show significant immunological predictors, late infections appeared to be influenced by T-cell lymphopenia and hypogammaglobulinemia. Our findings highlight potential factors that could guide strategies for managing post-transplant infections. These insights could contribute to more personalized approaches in immunosuppressive therapy.

## Data Availability

The original contributions presented in the study are included in the article/**Supplementary Material**. Further inquiries can be directed to the corresponding author.

## Supplementary material

Supplementary Figure 1 Distribution of CD4^+^ T naïve (Tn), effector (Teff), central memory (Tcm) and effector memory (Tefm) subsets in a cohort of pediatric liver recipients grouped by age ranges across each follow-up period: pre-transplantation (PreTx) and 1, 3, 6, 9 and 12 months post-transplantation (1M, 3M, 6M, 9M and 12M, respectively).

Supplementary Figure 2 Distribution of CD8^+^ T naïve (Tn), effector (Teff), central memory (Tcm) and effector memory (Tefm) subsets in a cohort of pediatric liver recipients grouped by age ranges across each follow-up period: pre-transplantation (PreTx) and 1, 3, 6, 9 and 12 months post-transplantation (1M, 3M, 6M, 9M and 12M, respectively).
