# Supplementary figures and images for: Immune monitoring and risk of infection in pediatric liver transplantation: a prospective study
Source: Front Immunol. 2025 Jun 12;16:1605716. doi: 10.3389/fimmu.2025.1605716 (PMC12197944; doi:10.3389/fimmu.2025.1605716)

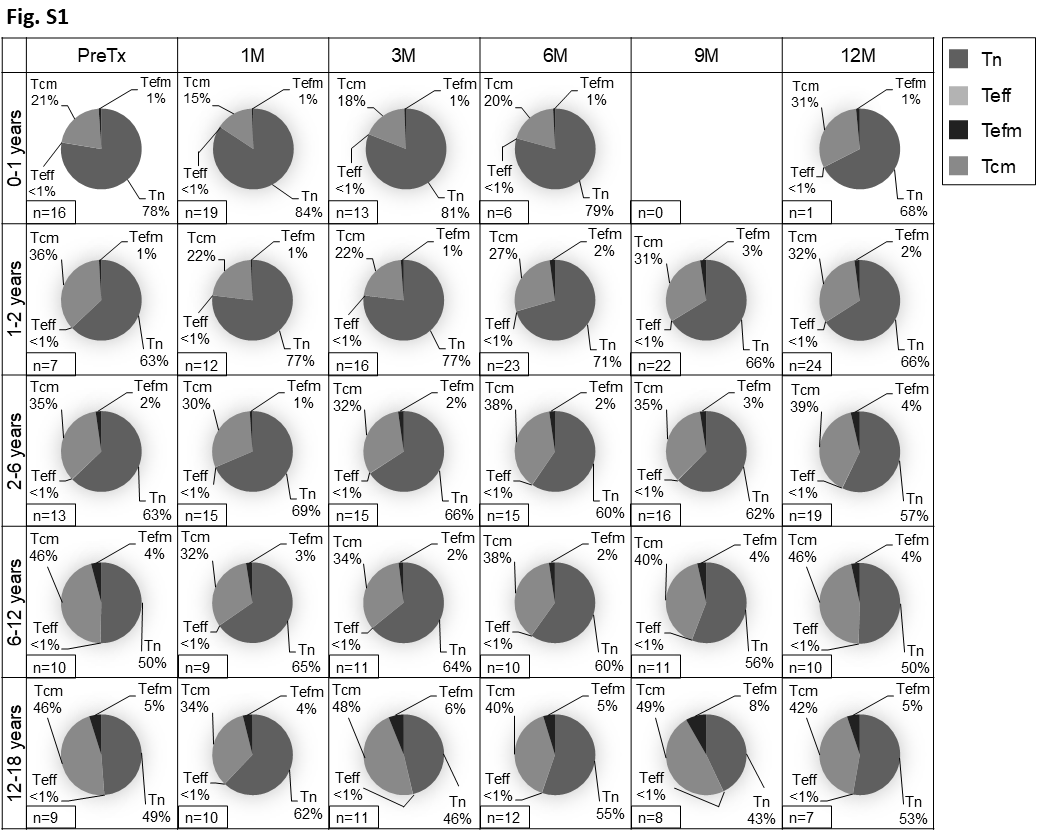

Supplement: Supplementary Figure 1 — Distribution of CD4+ T naïve (Tn), effector (Teff), central memory (Tcm) and effector memory (Tefm) subsets in a cohort of pediatric liver recipients grouped by age ranges across each follow-up period: pre-transplantation (PreTx) and 1, 3, 6, 9 and 12 months post-transplantation (1M, 3M, 6M, 9M and 12M, respectively). [file Image1.tif]

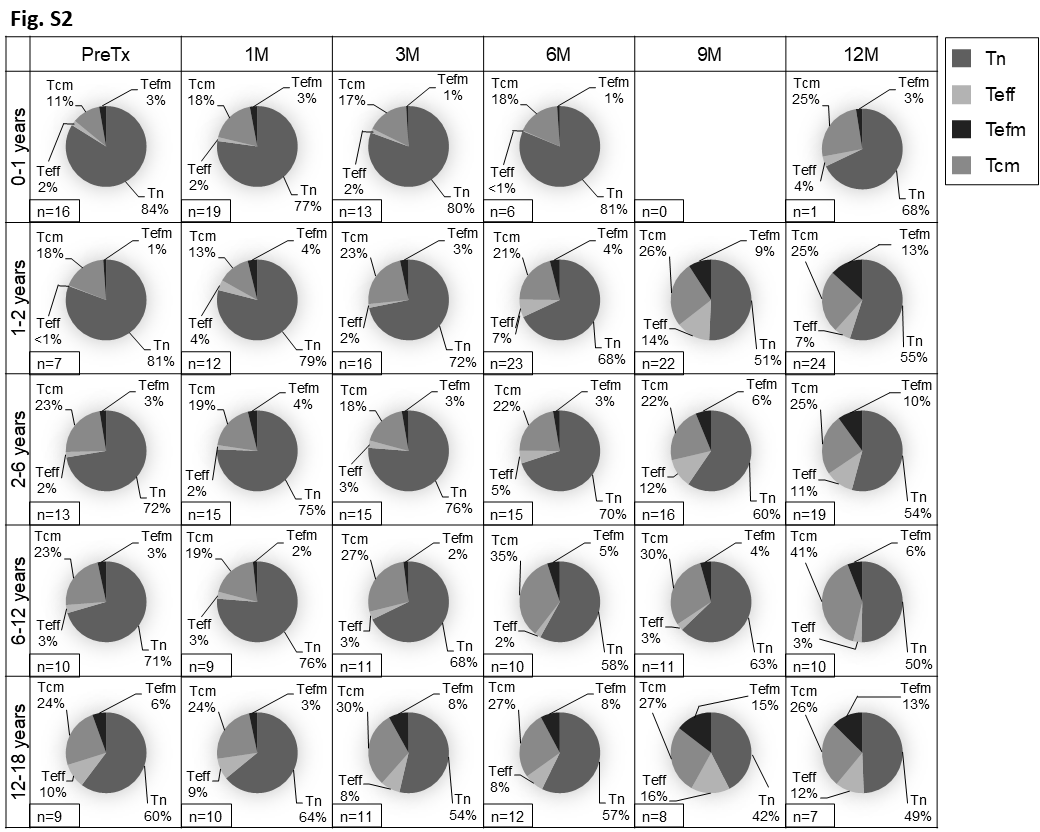

Supplement: Supplementary Figure 2 — Distribution of CD8+ T naïve (Tn), effector (Teff), central memory (Tcm) and effector memory (Tefm) subsets in a cohort of pediatric liver recipients grouped by age ranges across each follow-up period: pre-transplantation (PreTx) and 1, 3, 6, 9 and 12 months post-transplantation (1M, 3M, 6M, 9M and 12M, respectively). [file Image2.tif]
