# Supplementary material for: Immune monitoring and risk of infection in pediatric liver transplantation: a prospective study
Source: Front Immunol. 2025 Jun 12;16:1605716. doi: 10.3389/fimmu.2025.1605716 (PMC12197944; doi:10.3389/fimmu.2025.1605716)
Supplement: Supplementary file 3 [file DataSheet1.docx]

**Table S1.** **Panel of flow-cytometry antibodies used during the whole recruitment period in a cohort of pediatric liver recipients.**

| **FC panel** | | **Fluorochromes** | | | | | | | | | | | | |
| --- | --- | --- | --- | --- | --- | --- | --- | --- | --- | --- | --- | --- | --- | --- |
|  |  | **FITC** | **PE** | **ECD** | **PerCP (BD) / PC5.5 (BC)** | **PeCy7** | **APC** | **APC-H7 (BD) / APC-AF750 (BC)** | **APC-AF700** | **BV421 (BD) / PB (BC)** | **BV510 (BD) / KO (BC)** | **BV605 (BD)** | **BV650 (BD)** | **BV786 (BD)** |
| **From January 2019 to December 2020 (BD)** | **Tube 1** | CD3  (clone UCHT1) | CD16  (clone 3G8) CD56  (clone NCAM16.2) |  | CD4  (clone SK3) | CD19  (clone SJ25C1)  TCRγδ  (clone 11F2) | CD45  (clone HI30) | CD45RO  (clone UCHL1) |  | CD27  (clone M-T271) | CD8  (clone SK1) |  |  |  |
|  | **Tube 2** | HLA-DR  (clone G46-6) | CD31  (clone WM59) |  | CD4  (clone SK3) | CD127  (clone HIL-7R-M21) | CD25  (clone 2A3) | CD45RA  (clone 5H9) |  | CD3  (clone UCHT1) | CD8  (clone SK1) |  |  |  |
| **From January 2021 to May 2021 (BC)** | **Tube 1** | CD45RO  (clone UCHL1) | CD31  (clone 1F11) | CD3  (clone UCHT1) | CD27  (clone 1A4CD27) | CD19  (clone J3-119)  TCRγδ  (clone IMMU510) | CD16  (clone 3G8)  CD56  (clone N901) | CD4  (clone 13B8.2) | CD8  (clone B9.11) | HLA-DR  (clone Immu357) | CD45  (clone J33) | CD45RA  (clone HI100) | CD25  (clone M-A251) | CD127  (clone HIL-7R-M21) |
| **From June 2021**  **to July 2024 (BC)** | **Tube 1** | CD45RO  (clone UCHL1) | CD27  (clone 1A4CD27) | CD3  (clone UCHT1) |  | CD19  (clone J3-119)  TCRγδ  (clone IMMU510) | CD16  (clone 3G8)  CD56  (clone N901) | CD4  (clone 13B8.2) | CD8  (clone B9.11) |  | CD45  (clone J33) |  |  |  |
|  | **Tube 2** | CD25  (clone B1.49.9) | CD31  (clone 1F11) | CD3  (clone UCHT1) |  | CD45RA  (clone 2H4) | CD127  (clone R34.34) | CD4  (clone 13B8.2) | CD8  (clone B9.11) | HLA-DR  (clone Immu357) | CD45  (clone J33) |  |  |  |

BC, Beckman Coulter S.L.; BD, Beckton Dickinson Biosciences S.A.; FC, flow-cytometry; FITC, Fluorescein isothiocyanate; PE, Phycoerythrin; PeCy7, Phycoerythrin-Cyanine 7; APC, Allophycocyanin; APC-AF700, Allophycocyanin-Alexa Fluor 700; APC-AF750, Allophycocyanin-Alexa Fluor 750; APC-H7, Allophycocyanin-Hilite 7; BV421, Brilliant Violet 421; BV510, Brilliant Violet 510; BV600, Brilliant Violet 600; BV650, Brilliant Violet 650; BV780, Brilliant Violet 780; ECD, Energy Coupled Dye (Phycoerythrin-Texas Red); FITC, Fluorescein isothiocyanate; KO, Krome Orange; PB, Pacific Blue; PC5.5, Peridinin-Chlorophyll 5.5; PerCP, Peridinin-Chlorophyll; PeCy7, Phycoerythrin-Cyanine 7; PE, Phycoerythrin

**Table S2. Pathogens responsible for early and late infections in a cohort of pediatric liver recipients, categorized by bacteria, fungi, and viruses.**

| Early infections (n=41) | | Number of patients, n |
| --- | --- | --- |
| Bacterial infection | *Klebsiella pneumoniae* | 4 |
|  | *Enterobacter cloacae* | 2 |
|  | *Enterococcus faecalis + Escherichia coli* | 2 |
|  | *Escherichia coli* | 2 |
|  | *Bacteroides fragilis* | 1 |
|  | *Bacteroides vulgatus* | 1 |
|  | *Chryseobacterium sp.+ Stenotrophomonas maltophilia* | 1 |
|  | *Clostridium difficile* | 1 |
|  | *Elizabethkingia meningoseptica + Klebsiella pneumoniae* | 1 |
|  | *Enterococcus cecorum* | 1 |
|  | *Enterococcus faecalis + Enterococcus gallinarum + Escherichia coli* | 1 |
|  | *Enterococcus faecalis + Serratia marcescens* | 1 |
|  | *Enterococcus gallinarum* | 1 |
|  | *Escherichia coli + Enterococcus avium* | 1 |
|  | *Escherichia coli + Klebsiella pneumoniae* | 1 |
|  | *Klebsiella variicola* | 1 |
|  | *Stenotrophomonas maltophilia* | 1 |
| Fungal infection | *Candida parapsilosis* | 5 |
|  | *Trichosporon inkin* | 2 |
| Fungal + bacterial infection | *Candida albicans + Enterococcus faecalis* | 1 |
| Viral infection | Rhinovirus | 2 |
|  | SARS-CoV-2 | 2 |
|  | Adenovirus | 1 |
|  | Astrovirus | 1 |
|  | Bocavirus + HCoV-OC43 | 1 |
|  | HCoV-NL63 | 1 |
|  | Norovirus | 1 |
|  | Rotavirus | 1 |
| Late infections (n=39) | |  |
| Bacterial infection | *Klebsiella pneumoniae* | 3 |
|  | *Clostridium difficile* | 2 |
|  | *Escherichia coli* | 2 |
|  | *Escherichia coli + Klebsiella pneumoniae* | 2 |
|  | *Enterococcus faecalis* | 1 |
|  | *Enterococcus faecium* | 1 |
|  | *Stenotrophomonas maltophilia* | 1 |
| Viral infection | SARS-CoV-2 | 6 |
|  | Rotavirus | 5 |
|  | Influenzavirus A | 3 |
|  | Norovirus | 3 |
|  | Rinovirus | 3 |
|  | Epstein-Barr | 2 |
|  | Adenovirus | 1 |
|  | Adenovirus + Norovirus | 1 |
|  | Enterovirus + Rinovirus | 1 |
|  | Norovirus + Rhinovirus | 1 |
|  | Norovirus + Rinoviurs + Sapovirus | 1 |

HCoV-NL63, Human coronavirus NL63; HCoV-OC43, Human coronavirus OC43; SARS-CoV-2, Severe Acute Respiratory Syndrome Coronavirus 2

**Table S3. Number of pediatric liver recipients with lymphopenia or hypogammaglobulinemia at each timepoint of the follow-up.**

| **Lymphopenia, n (%)** | **PreTx** | **1M** | **3M** | **6M** | **9M** | **12M** |
| --- | --- | --- | --- | --- | --- | --- |
|  | **n=79** | **n=89** | **n=85** | **n=82** | **n=67** | **n=64** |
| **Total** | 63 (78) | 32 (36) | 30 (35) | 23 (28) | 24 (36) | 9 (14) |
| **T cell CD3^+^** | 64 (81) | 33 (37) | 24 (28) | 18 (22) | 13 (19) | 3 (5) |
| **T cell CD3^+^CD4^+^** | 64 (81) | 26 (29) | 28 (33) | 22 (27) | 25 (37) | 9 (14) |
| **T cell CD3^+^CD8^+^** | 60 (76) | 28 (31) | 20 (24) | 18 (22) | 13 (19) | 4 (6) |
| **B cell CD19^+^** | 33 (42) | 10 (11) | 13 (15) | 16 (20) | 14 (21) | 9 (14) |
| **NK cell CD3^-^CD16^+^CD56^+^** | 5 (6) | 0 (0) | 3 (4) | 1 (1) | 0 (0) | 0 (0) |
| **Hypogammaglobulinemia, n (%)** | **PreTx** | **1M** | **3M** | **6M** | **9M** | **12M** |
|  | **n=76** | **n=89** | **n=85** | **n=82** | **n=67** | **n=64** |
| **IgG** | 0 (0) | 18 (20) | 15 (18) | 16 (20) | 5 (7) | 2 (3) |
| **IgA** | 2 (3) | 19 (21) | 18 (21) | 11 (13) | 2 (3) | 2 (3) |
| **IgM** | 0 (0) | 17 (19) | 22 (26) | 19 (23) | 6 (9) | 7 (11) |

1M, 1 month post-transplantation; 3M, 3 months post-transplantation; 6M, 6 months post-transplantation; 9M, 9 months post-transplantation; 12M, 12 months post-transplantation; Ig, immunoglobulin; PreTx, pre-transplantation**.**

**Table S4. Lymphocyte subpopulation scores in a cohort of pediatric liver recipients segregated according to their indication for transplantation.**

| **Score** | **Cholestasis /biliary atresia** | **Metabolic diseases** | **Liver tumours** | **Cirrhosis (other)** | **Severe acute liver failure** |  |
| --- | --- | --- | --- | --- | --- | --- |
|  | **(n=50)** | **(n=12)** | **(n=8)** | **(n=6)** | **(n=3)** | **p-value** |
| **Lymphocytes** | -0.81  (-0.96 to -0.56) | -0.80  (-0.89 to -0.24) | -0.63  (-0.89 - to -0.37) | -1.06  (-1.08 to -1.03) | -0.73  (-0.87 to -0.68) | 0.192 |
| **T-cell CD3^+^** | -0.76  (-0.93 to -0.60) | -0.73  (-0.81 to -0.44) | -0.73  (-0.91 - to -0.25) | -1.10  (-1.15 to -0.98) | -0.71  (-0.80 to -0.66) | 0.120 |
| **T-cell CD3^+^CD4^+^** | -0.74  (-0.86 to -0.58) | -0.68  (-0.84 to -0.36) | -0.67  (-0.86 - to -0.17) | -0.97  (-1.01 to -0.89) | -0.76  (-0.82 to -0.67) | 0.192 |
| **T-cell CD3^+^CD8^+^** | -0.71  (-0.79 to -0.53) | -0.70  (-0.80 to -0.44) | -0.53  (-0.65 - to -0.30) | -0.82  (-0.85 to -0.81) | -0.55  (-0.64 to -0.53) | 0.109 |
| **B-cell CD19^+^** | -0.43  (-0.57 to -0.22) | -0.52  (-0.60 to -0.25) | -0.52  (-0.62 - to -0.07) | -0.58  (-0.70 to -0.50) | -0.38  (-0.46 to -0.15) | 0.446 |
| **NK-cell CD3^-^CD16^+^CD56^+^** | -0.34  (-0.44 to -0.21) | -0.30  (-0.33 to -0.21) | -0.11  (-0.34 - to 0.19) | -0.44  (-0.46 to -0.40) | -0.40  (-0.44 to -0.39) | 0.056 |
